# Relationship between enunciative signs of language acquisition and language assessment through the Bayley III scale at 24 months

**DOI:** 10.1590/2317-1782/20232021221en

**Published:** 2023-05-15

**Authors:** Luciéle Dias Oliveira, Anaelena Bragança de Moraes, Sabrina Felin Nunes, Inaê Costa, Ana Paula Ramos de Souza

**Affiliations:** 1 Programa de Pós-graduação em Distúrbios da Comunicação Humana, Departamento de Fonoaudiologia, Universidade Federal de Santa Maria - UFSM - Santa Maria (RS), Brasil.; 2 Programa de Pós-graduação em Distúrbios da Comunicação Humana, Departamento de Estatística, Universidade Federal de Santa Maria - UFSM - Santa Maria (RS), Brasil.; 3 Departamento de Saúde da Comunicação Humana, Instituto de Psicologia, Universidade Federal do Rio Grande do Sul - UFRGS - Porto Alegre (RS), Brasil.; 4 Programa de Pós-graduação em Distúrbios da Comunicação Humana, Universidade Federal de Santa Maria - UFSM - Santa Maria (RS), Brasil.

**Keywords:** Language, Development, Risk Factors, Childhood, Assessment

## Abstract

**Purpose:**

To analyze the correlation between the results obtained on the SEAL and the Bayley III Scale and compare babies with and without delay in language acquisition at 24 months concerning the performance obtained by them and their mothers on the SEAL from 3 to 24 months.

**Methods:**

The SEAL collection consists of 15-minute footages of 45 babies aged from 3 to 24 months old in interaction with their mothers, who were assessed by two trained speech therapists for the use of the SEAL. At 24 months, the 45 babies were assessed using the Bayley III Scale and the item language was selected to classify them with and without delay. These results were statistically analyzed through a Pearson’s correlation test and a Fisher's exact test.

**Results:**

In average, eighteen signs of typical development as we obtained, while a mean of 12 delay signs were found. By comparing the presence and absence of signs between the groups with and without delay in language acquisition, eight signs from the baby and one from the mother differed statistically in the sample. The analysis using the SEAL for cases of delay showed that the maternal factor was as important as the infant factor to understand the babies’ language functioning.

**Conclusion:**

There was a significant correlation between the SEAL performance from 3 to 24 months and the language outcome at 24 months assessed by the Bayley III Scale in this sample.

## INTRODUCTION

Infant language acquisition should be assessed in the ealry years of life since there is specialized childcare monitoring that analyzes this developmental aspect. The Bayley III Scale^(1)^ is one of the instruments that allows for this assessment in the first two years of life, considered the gold-standard examination for infant development assessment and widely used by the scientific community^(2-10)^ for differentiating receptive communication (49 items) from expressive communication (48 items) in the infant grammar domain.

According to Madaschi et al.^(11)^ in a study on cross-cultural adaptation and psychometric properties, the Brazilian version of the Bayley III scale showed a highly convergent validity, as well as good internal consistency and homogeneity of items for children aged 12 to 42 months, thus corroborating its effectiveness for research purposes.

Although this scale has diagnostic value for the grammatical domain, it involves some application time (one to two sessions) and depends on the collaboration of the child, as well as on specialized training by the examiner and acquisition of high-cost materials in the context of the common reality of Brazilian professionals. Furthermore, it does not investigate the adult's participation in the language acquisition process.

The Enunciative Signs of Language Acquisition [ESLA - in Portuguese *Sinais Enunciativos de Aquisição da Linguagem* (SEAL)]^(12-14)^ tool was preliminarily validated to provide an instrument to address the adult-child dialogue so that it could be easily applied in the process of language acquisition follow-up, based on the contributions of clinical studies from the enunciative perspective^(15-18)^ and the enunciative study of language acquisition^(19)^. The ESLA signs consider the semiotic level (grammatical domain of the language) and the language semantization process. This process is related to the subject's appropriation of their linguistic knowledge (semiotic level) in the dialogue support, which allows for identifying the emergence and support of a place of enunciation for the baby^(16-19)^.

From this perspective, the child's potentialities (biological, cognitive, and subjective) and the enunciative support offered by the adult are important^(17,18)^. The ESLA signs capture whether the language acquisition process is proceeding as expected or it presents some impediment through an indicative paradigm, *i.e*., if the signs are present, the process is possibly satisfactory; if they are absent, the child and their family members should be monitored through shorter sessions to verify the language progress and establish a potential demand for timely intervention. ESLA studies indicate a child factor and a maternal factor in the language functioning between babies and their mothers, which contributes to the understanding of obstacles to language acquisition. ESLA is not aimed at diagnosis but at monitoring language acquisition.

In this context, herein we consider the results from previous studies on the grammatical domain and the language semantization process. The goal is to analyze the child’s conditions of occupying the enunciation place, as well as the adult’s conditions of sustaining this place^(12-19)^, in addition to the scientific evidence generated by the Bayley Scale III^(1-11)^. Thereby, this research analyzes the correlation between the results obtained by the ESLA and the Bayley Scale III, and compares babies with and without delayed language acquisition at 24 months of age concerning their performance and their mothers’ in the ESLA from 3 to 24 months.

## METHODS

This is a quantitative, longitudinal, and prospective study that was approved by the Research Ethics Committee of an educational institution in a medium-sized city in the Rio Grande do Sul state - CAAE number: 28586914.0.0000.5346. This study is in line with the regulatory norms and guidelines for research with human beings established in Resolution 466/12 of the National Health Council. It provides for data confidentiality, thus ensuring both the secrecy and privacy of the subjects’ identities by the signing of the Confidentiality Agreement. In addition, the families who agreed to participate in the research and signed the Free and Informed Consent Form (FICF) were instructed on the objectives and procedures to the research. Those responsible for the babies answered the interview on sociodemographic, obstetric, and psychosocial data adapted from the original version by Schwengber and Piccinini^(20)^.

Initially, the sample for the ESLA assessment included 101 babies, out of which only 45 children remained, 19 born at term and 26 pre-terms, followed by the ESLA from 3 to 24 months, who were assessed at 24 months based on the Bayley Scale III. Our research analyzes the language item, all other developmental aspects were analyzed based on the Bayley Scale III by other professionals participating in a larger research. For babies born prematurely, the corrected age was considered in the assessment. The babies and their families were invited to participate in the research during the follow-up sessions of premature babies at a university hospital, and during the Guthrie test at a Primary Health Care Unit nearby. The following inclusion criteria were applied: babies without biological limitations, such as neurological lesions or syndromes, or sensory deficits (visual, auditory, etc.). These aspects were assessed by pediatricians and the research team, including speech therapists, psychologists, physiotherapists, and occupational therapists. In case of doubt, they were removed from the study and referred to a neuro pediatrician or geneticist.

The language analysis based on ESLA involves filming the interaction of the mother, or whoever performed this function for the baby, which occurred in different ways throughout the research stages. The filming was carried out from two angles, frontal and lateral, encompassing a time of 15 minutes, depending on the baby’s age and other aspects to be analyzed. The babies were positioned in a baby-comfort or seated on an EVA mat to interact with their mothers in a lighted and comfortable environment in terms of temperature. They should be in a good state of wakefulness, well fed, and sanitized. The filming was performed using two JVC Everio GZ-MG 630 camcorders placed in two positions: two meters away from the mother and the baby, with the mother positioned with her back to the camcorder and the baby in front, and another placed one meter away, with the mother and the baby interacting face to face at a side angle.

The postures were standardized so that the baby would be observed interacting with their mother. The babies were born at term or were late preterm infants, in the case of the latter, the corrected age was considered.

The babies filmed for the ESLA analysis, according to the four six-monthly instruments created^(12-14)^, were dividied into the following age groups:

**Phase 1 - 3 months and 1 day to 4 months and 29 days** - The baby sitting in the baby carrier (9 minutes). The mother was instructed to sing (3 minutes) (ambiance), talk to the baby (3 minutes), and offer an object - *e.g*., a rubber dog without noise (3 minutes).
**Phase 2 - 8 months and 1 day to 9 months and 29 days** - Mother and baby seated on the EVA mat were filmed in the interaction, and the mother was asked to sing to the baby for 3 minutes, talk for another 6 minutes, and play with an object (the rubber dog) offered by the examiner (6 minutes). If the baby did not have trunk control, they could use a comfort baby.
**Phase 3 - 17 months and one day to 18 months and 29 days and Phase 4 - 23 months and one day to 24 months and 29 days** - At these phases, the baby was observed in free activity with the mother playing with a box of thematic toys (animals, a baby with a bottle, small pans, etc.), in addition to plays and linguistic interactions between the mother and the baby. The mother was instructed to remain on the EVA mat with the baby during the filming. Over the first 10 minutes, the interaction of the mother with the baby was filmed and in the last 5 minutes, the examiner participated in the interaction to observe some signs that covered the dialogue with different interlocutors.

The videos were watched by two qualified speech therapists who assigned the signs of the ESLA instruments to the babies, thus allowing for verifying an agreement of 95 and 100% between both of them. Our analysis considered, the values assigned by the main researcher, the main author of this article.

Table 1 summarizes the enunciative signs analyzed.

**Table 1:** Enunciative Signs of Language Acquisition (ESLA)

**Signs from 2 to 6 months and 29 days**	**Analyzed speaker**
1. The child reacts to motherese, through vocalizations, body movements, or looks.	baby
2. The child fills its place in the interlocution with verbal sounds such as vowels and/or consonants.	baby
**3. The child fills her place in the interlocution with non-verbal sounds in tune with the enunciative context (smile, cry, cry, cough, grumble).**	**baby**
**4. The child fills his place in the interlocution silently only with body movements and looks attuned to the enunciative context.**	**baby**
5. Child initiates conversation or proto-conversation.	baby
6. The child and the mother (or her substitute) exchange glances during the interaction.	baby-mother
**7. The mother (or her substitute) assigns meaning to the baby's verbal and non-verbal manifestations and sustains this proto-conversation or conversation when the baby initiates it.**	**mother**
8. The mother (or her substitute) uses motherese by talking to the child in a way that is attuned to what is happening in the context and waiting for the baby's responses.	mother
**Signs from 7 to 12 months and 29 days**	
9. The child fills its place in the interlocution (enunciation) with verbal sounds (syllables with varied vowels and consonants - at least two points and two consonant articulatory modes).	baby
10. The child outlines the production of proto words by mirroring the mother's (or substitute's) speech.	baby
**11. Child outlines the production of proto words spontaneously.**	**baby**
**12. When the mother (or substitute) is called upon to enunciate by the child, she produces her enunciation and waits for the child's answer.**	**mother**
**Signs from 13 to 17 months and 29 days**	
**13. The child names spontaneously and intelligibly to the adult interlocutor, objects that are absent in the context.**	**baby**
**14. The child names in a spontaneous way, but not intelligible to the interlocutor adult, objects that are absent in the context, seeking in the prosody a way to be understood.**	**baby**
**15. The child names in a spontaneous and intelligible way to the interlocutor adult, objects, people, and actions, which are present in the enunciative context.**	**baby**
**16. The child makes gestures to try to make himself/herself understood when the adult interlocutor does not understand him/her.**	**baby**
**17. The child repeats what the adult interlocutor says as a way of zorganizing or reorganizing his or her utterance, for example by improving the syntactic or phonological form, the choice of the lexical item, or even by accentuating some item prosodically.**	**baby**
**18. The child talks to different adult interlocutors (father, mother, examiner).**	**baby**
**19. The adult interlocutor attributes a possible meaning to the child's verbal productions, that is, in a tuned way.**	**mother**
**Signs 18 to 24 months and 29 days**	
**20. The child requests objects and/or asks for clarifications from the interlocutor adult, marking his position as speaker.**	**baby**
**21. The child uses distinct phonemic forms to convey different meanings in his/her utterance (at least two articulatory points - labial and alveolar - and two distinct consonantal sound classes - at least nasal and plosive).**	**baby**
**22. The child uses different forms (words) to convey different meanings in his/her enunciation.**	**baby**
**23. The child combines words, in direct or inverted form, to convey different meanings.**	**baby**
**24. When the child presents verbal productions distinct from adult speech, the adult interlocutor reacts by making a neutral repair request (what) or by correctly repeating the child's speech, or offering a lexical item compatible with the infant's communicative intention.**	**mother**

Source: Crestani et al.^(12,13)^, Fattore et al.^(14)^. Significant signs in the factor analysis are in bold

All subjects were also assessed at phase 4 (aged 24 months) using the Bayley Scale III^(1,11)^ by a qualified professional. Herein, the language subscale (receptive communication and expressive communication) was considered. Initially, the starting point was found in the test of each baby based on their age. The assessment started as soon as the baby consecutively scored the first three questions (basepoint) and ended upon five consecutive errors.

The statistical analysis used an Excel database to organize the language data generated from the presence and absence of ESLA signs in each age group and total, as well as the Bayley III scores obtained for 24 months. All statistical analyses of the results were performed on the STATISTICA 9.1 software. Herein we consider the significance level of *p* ≤ 0.05. Pearson's correlation and Fisher's exact test were also used.

## RESULTS

We analyzed forty-five infants and verified the correlation between the total number of ESLA signs obtained through the four six-monthly instruments and the language scores generated using the Bayley Scale III at 24 months. The results scored a **Pearson correlation of 0.718 and a *p* value of 0.001**, thus indicating statistical significance (*p*<0.05). It allows us to infer that the higher the ESLA score the higher the Bayley III score.

The comparative analysis between children with and without language delay, based on the results from the language assessment by the Bayley Scale III at 24 months, showed the average number of enunciative signs of language acquisition in each group, as shown in Table 2.

**Table 2:** Comparison of ESLA total score *versus* Bayley scale III

TESTS	WITHOUT RISK (LANGUAGE) Bayley III at 24 months				WITH RISK (LANGUAGE) Bayley III at 24 months				*p*_value
	N	Mean (±SD)	Minimum	Maximum	N	Mean (±SD)	Minimum	Maximum	
TOTAL ESLA	25	18.85 (± 2.92)	15.00	22.00	20	12.39 (± 5.13)	2.00	22.00	0.001*

* Significant by Mann-Whitney U test

**Caption:** ESLA = Enunciative Signs of Language Acquisition; SD = Standard Deviation; N = Number of Subjects

On average, the babies with no delay in language acquisition presented 18 signs, whereas the babies with delay presented 12 signs, *i.e.,* which reflected on the difference between both groups when comparing the absence and presence of each sign, as shown in Table 3.

**Table 3:** Comparative analysis by a sign of the babies with and without language delay according to the Bayley III scale

SIGNS ESLA	Babies with delay n=20				Babies without delay n=25				Comparison*
	Dyads with missing signs		Dyads with present signs		Dyads with missing signs		Dyads with present signs		
	n	%	n	%	N	%	n	%	*p*-value
1 (B)	2	10	18	90	2	8	23	92	0.606
2 (B)	6	30	14	70	3	12	22	88	0.131
**3 (B)**	2	10	18	90	2	8	23	92	0.606
**4 (B)**	2	10	18	90	2	8	23	92	0.606
5 (B)	10	50	10	50	11	44	14	56	0.460
6 (B)	3	15	17	85	2	8	23	92	0.392
**7 (A)**	2	10	18	90	1	4	24	96	0.415
8 (A)	5	25	15	75	3	12	22	88	0.229
9 (B)	11	55	9	45	6	24	19	76	0.034*
10 (B)	13	65	7	35	7	28	18	72	0.014*
**11 (B)**	11	55	9	45	9	36	16	64	0.165
**12 (A)**	2	10	17	85	3	12	22	88	0.632
13 (B)	20	100	0	0	22	88	3	12	0.625
**14 (B)**	20	100	0	0	25	100	0	0	1.000
**15 (B)**	15	75	5	25	4	6	21	94	0.0001*
16 (B)	9	45	11	55	0	0	25	100	0.0002*
**17 (B)**	16	80	4	20	4	6	21	94	0.0000*
**18 (B)**	14	70	6	30	3	12	22	88	0.0001*
**19 (A)**	11	55	9	45	7	28	18	72	0.063
**20 (B)**	8	40	12	60	4	6	21	94	0.071
**21 (B)**	12	60	8	40	1	4	24	96	0.0000*
**22 (B)**	17	85	3	15	4	6	21	84	0.0000*
**23 (B)**	15	75	5	25	4	6	21	84	0.0001*
**24 (A)**	10	50	10	50	1	4	24	96	0.0005*

* Significant Fisher's exact test

**Caption:** Bold = Important Signs in Factor Analysis (Crestani et al.^(12,13)^, Fattore et al.^(14)^); B = Infant Shows Sign; A = Adult Shows Sign; n = Number of Infants; ESLA = Enunciative Signs of Language Acquisition

The signs 9, 10, 16, 17, 18, 21, 22, and 23, related to the infant’s enunciative aspects, and sign 24, related to the maternal position in the last age group, differed between the group with language acquisition delay and the group without delay, in terms of the sample statistical comparison . These signs were more markedly present in the babies with typical development than in the babies with delay.

Table 4 indicates the data descriptive analysis from the 20 children who presented language alteration according to the Bayley Scale III, at 24 months, indicating both the present and absent signs.

**Table 4:** Descriptive analysis of Enunciative Signs of Language Acquisition (ESLA) in the cases of language delay in Bayley III at 24 months

Subject	Present Signs ESLA	Absent Signs ESLA	Total ESLA Present	Bayley III at 24m
S1	1,2,3,4,5,6,7,8,12,16,18,19,20, 21,24	9,10,11, 13, 14, 15,17, 22,23	15	79 (B)
S2	1,3,4,5,6,7,8,16,18,	2,9,10,11,12, 13,14,15,17 19, 20,21,22,23,24	9	71 (B)
S3	1,2,3,4,5,6,7,8,9,10,11,12,	13,14,15,16,17,18,19,2021,22,23, 24	12	59 (EL)
S4	1,3,4,6,7,8,12, 16,19	2,5,9,10,11,13,14,15,17,18, 20,21,22,23,24	9	77 (B)
S5	1,2,3,4,5,6,7,8,9,10,11,12, 15,16,18,19,20,21,24	13,14,17,22,23	19	83 (LM)
S6	1,2,3,4,5,6,7,8,12, 20,21,24	9,10,11,13,14,15,16,17, 18,19, 22,23	12	79 (B)
S7	6,12	1,2,3,4,5,7,8,9,10,11,13, 14,15,16,17,18,19,20,21,22,23,24	2	65 (EL)
S8	1,3,4,6,7,8,12,20,21,22,23,24	2,5,9,10,11,13,14,15,16, 17,18,19	12	47 (EL)
S9	1,2,3,4,5,6,7,8,9,10,11,12,16,19,20,21,22	13,14,15,17,18,23,24	17	85 (LM)
S10	1,2,3,4,7,11,12,	5,6,8,9,10,13,14,15,16,17,18,19,20,21,22,23,24	7	59 (EL)
S11	1,2,3,4,5,6,10,11,12,20,21,22,23	7,8,9,13,14,15,16,17,18,19,24	13	74 (B)
S12	1,2,3,4,5,6,7,8,9,10,11,12,15,16,17,18,19,20,21,22,23,24	13,14	22	77 (B)
S13	1,2,3,4,7,9,11	5,6,8,10,12,13,14,15,16,17,18,19,20,21,22,23,24	7	47 (EL)
S14	2,7,8,15,16,17,18,19,20,21,22,24	1,3,4,5,6,9,1011,12,13,14,23	12	79 (B)
S15	1,3,4,6,7,12,16,20,21,24	2,5,8,9,1011,13,14,15,17,18,19,22,23	10	79 (B)
S16	1,2,3,4,5,6,7,8,9,10,11,12,16,	13,14,15,17,18,19,20,21,22,23,24	13	77 (B)
S17	1,2,3,4,6,7,8,9,12,15,16,17,18,19,20,21,22,23,24	5,10,11,13,14	19	79 (B)
S18	1,2,3,4,5,6,7,8,9,10,11,12,15,16,17,18,19,20,21,22,23,24	13,14	22	83 (LM)
S19	1,3,4,6,7,8,12,13,20,21	2,5,9,10,11,14,15,16,17,18,19, 22,23,24	10	83 (LM)
S20	1,2,3,4,6,7,8,9,12,20,21,22,23,24	5,10,11,13,14,15,16,17,18,19	14	89 (LM)

**Caption:** B = Borderline; EL = Extremely Low; LM = Low Medium; ESLA = Enunciative Signs of Language Acquisition

The children with alterations according to the ESLA reached borderline, low, or very low scores on the Bayley Scale III. The children who developed some delay in the assessment by the Bayley Scale III at 24 months showed altered child’s signs (child factor) and signs related to the maternal activity of dialogue support (maternal factor). Furthermore, from this group of 20 children with alteration by the Bayley Scale III, four were not at risk in the ESLA, according to the average number of signs (Table 2), nor did they present any alterations in the maternal factor (signs in bold). The four children without risk in the ESLA but with delay by the Bayley Scale III were assessed between borderline and low average. None of them received an extremely low rating.

Another relevant aspect in Table 4 is associated with the varying scores from the Bayley Scale III (borderline, very low, extremely low), highlighting most children in the extremely low category, a greater indication of severe delay, with the lowest values in the ESLA (2 to 7), except for subject 3 (S3) with 12 signs. However, a low value shows an agreement at least regarding the aspects concerning the child factor between both tests. The children within the other categories (borderline or very low) scored values between 9 and 17 signs in the ESLA.

## DISCUSSION

The positive correlation between cases of language delay assessed by the Bayley III Scale and risk cases in the ESLA demonstrates the effectiveness of this examination as a screening test for the sample studied. Table 4 shows that 16 of the 20 cases assessed as delay by the Bayley III Scale had significant alterations in the ESLA.

In this research, the ESLA was assigned by filming during childcare follow-up sessions, although it may be analyzed by a qualified professional by observing the mother-baby interaction in an outpatient clinic, which would be less expensive in terms of time and cost for insertion in the Universal Health System [in Portuguese *Sistema Único de Saúde* (SUS)]. In this condition, the suspected cases according to the ESLA would be referred to a diagnostic test through the Bayley Scale III or other diagnostic scales for language and development^(21,22)^. The number of mother-baby dyads participating in the ESLA investigation in all age groups was much higher (101) than in the group that attended the two assessment meetings for the Bayley Scale III (45). This sample loss suggests that the adherence to the more time-consuming test (the Bayley Scale III) by users demands a change of culture and an improvement in the access to the health service, which is not expected to change on a short-term perspective.

In contrast, the comparison between the group with and without delay allowed us to establish an average of 18 enunciative signs out of 24 assessed as the absence of risk in the ESLA. These data suggest the need to continue investigating the ESLA in terms of establishing criteria per age group and in the total test, which was not possible from the small sample obtained in this research.

Table 3 shows signs, such as the 13 and 14, that were absent in both groups, although sign 14 was indicated as relevant in the factor analysis^(14)^. Among the signs that statistically differed when comparing infants with and without delay, signs 9, 10, 21, 22, and 23 showed the ability of infants without language delay to occupy their place in enunciation with increasingly complex vocalization and speech (phonological and lexical diversity, and initial use of syntax). In turn, babies without these signs may show a potential delay in language acquisition.

Sign 16 is related to the use of gestures as a form of communication, which is predicted by language acquisition studies that claim some continuity and synchrony between verbal skills and baby gestures^(23)^.

Sign 17 is related to the baby's ability to anchor themselves in the mother's speech to improve what they say, a strategy identified by Silva^(19)^ in the language acquisition process. It is also related to an adult's willingness to help the child speaking, assessed in sign 24. Therefore, it is important to observe the strength of both signs simultaneously in the sample studied.

In addition to those already mentioned, sign 18, related to the amplitude of interlocutors, was fundamental to assess not only the disjunction in terms of the enunciative acquisition relation but also of the mother-baby separation process. Such a scenario was observed by Flores and Souza^(24)^, who found that babies in psychological suffering and facing difficulties in the separation process and operation of the paternal function showed difficulty in speaking with distinct interlocutors.

The lack of distinctive sign when comparing the two groups in the first age group (zero to six months) indicates the need for further investigations and improving the instrument. Likewise, the factor analysis showed three signs at this phase that were related to a child factor and a maternal factor in a larger sample of subjects at the same phase^(13)^. These results suggest the need for continuous studies on validation criteria.

Table 4 shows that subjects 5, 12, 17, and 19, presented 18 or more enunciative signs. It is worth mentioning that these four subjects presented no alterations in the mother factor, nor did subject 5, with delay according to both ESLA and Bayley Scale III, whereas all others did. They also did not obtain the “extremely low” classification in the Bayley Scale III^(1)^. These data allow us to observe that, in most cases, the maternal factor contributed to the emergence and understanding of language functioning in cases of delayed language acquisition. In other words, the way the adult carries out enunciative support must be considered in the assessment and intervention for delayed language acquisition^(17,18)^.

Several studies in the enunciative field^(12-19)^ have evidenced that both language acquisition and clinical practice with young children should invest in the analysis of the mother-baby dialogue to propose a hypothesis of language functioning^(16)^ that allows proposing intervention lines. This language operating hypothesis foresees the relation I (child) - YOU (adult) in the understanding of the suffering arising from language delay or disorder. Based on this theoretical perspective and our results, the ESLA is a promising tool for assessing such a factor, was as it was revealed in 16 out of 20 cases assessed as language delay by the Bayley III Scale. It is important highlighting that children with extremely low values on the Bayley III Scale were the same ones who received lower ESLA values.

Considering the numerical limitation of our sample, the results suggest the need to continue investigating the language of infants and young children using ESLA since it is an effective way to monitor language acquisition in childcare and propose interventions in time to prevent the crystallization of language symptoms^(17,18)^.

Such a scenario requires to establish criteria for the test in larger samples. Ours is a clinical study of a smaller proportion, which included babies who attended the assessment using the Bayley Scale III at the end of the research, at two years of age, a number much smaller than should be desired. ESLA assessments have no diagnostic purposes since the baby is undergoing the process of linguistic constitution, rather they seek to offer timely interventions to favor the convergence between family members and the baby or small child. In this context, facilitating the maternal factor, an important element in the factorial studies, is a way of strengthening the convergence and linguistic synchrony between the mother (or her substitute) and the baby. This shows that the field of speech therapy could benefit from studies centered on dialogue as the analysis focus in research on language acquisition since children's abilities to occupy their place of enunciation are as relevant in the acquisition process as the adult's support of an enunciative place.

## CONCLUSION

Our findings allow us to suggest a significant correlation between the performance in the ESLA between 3 and 24 months and the language outcome at 24 months assessed by the Bayley Scale III. The comparison between babies with and without delay in language acquisition allowed us to establish averages of signs in the ESLA. Additionally, some signs from the baby and one from the mother showed statistical differences when comparing the two groups of the sample studied as to their presence and absence, especially from the second age group studied.

These data allow us to conclude that the ESLA has some potential as a screening test and should be investigated in larger samples since it involves a short application time requiring only to observe the mother-baby interaction during the first and second years of life in a context of spontaneous play with materials that are accessible to examiners and families.
