# Supplementary material for: Relationship between enunciative signs of language acquisition and language assessment through the Bayley III scale at 24 months
Source: Codas. 2023 May 15;35(3):e20210221. doi: 10.1590/2317-1782/20232021221en (PMC10205087; doi:10.1590/2317-1782/20232021221en)
Supplement: Translation

# Translation: Relação entre sinais enunciativos de aquisição da linguagem e a avaliação de linguagem pela escala Bayley III aos 24 meses

**DOI:** 10.1590/2317-1782/20232021221pt

**Authors:** Luciéle Dias Oliveira, Anaelena Bragança de Moraes, Sabrina Felin Nunes, Inaê Costa, Ana Paula Ramos de Souza

**Affiliations:** 1 Programa de Pós-graduação em Distúrbios da Comunicação Humana, Departamento de Fonoaudiologia, Universidade Federal de Santa Maria - UFSM - Santa Maria (RS), Brasil.; 2 Programa de Pós-graduação em Distúrbios da Comunicação Humana, Departamento de Estatística, Universidade Federal de Santa Maria - UFSM - Santa Maria (RS), Brasil.; 3 Departamento de Saúde da Comunicação Humana, Instituto de Psicologia, Universidade Federal do Rio Grande do Sul - UFRGS - Porto Alegre (RS), Brasil.; 4 Programa de Pós-graduação em Distúrbios da Comunicação Humana, Universidade Federal de Santa Maria - UFSM - Santa Maria (RS), Brasil.

**Keywords:** Linguagem, Desenvolvimento Infantil, Fatores de Risco, Infância, Avaliação

## Abstract

**Objetivo:**

Analisar a correlação entre resultados obtidos no Sinais Enunciativos de Aquisição da Linguagem (SEAL) e na Escala Bayley III e comparar bebês com e sem atraso na aquisição da linguagem aos 24 meses no desempenho obtido por ele e sua mãe no SEAL dos 3 aos 24 meses.

**Método:**

A coleta do SEAL constou de filmagens de 45 bebês, realizadas nas faixas etárias de 3 a 24 meses em interação com suas mães, com duração de 15 minutos, que foram avaliados por duas fonoaudiólogas treinadas no uso do SEAL. Aos 24 meses, os 45 bebês foram avaliados pela Escala Bayley III e selecionado o item linguagem para classificá-los com e sem atraso. Sobre tais resultados realizaram-se as análises estatísticas com o teste de correlação de Pearson e o teste exato de Fisher.

**Resultados:**

Obtiveram-se as médias de sinais no desenvolvimento típico que foi 18 sinais e, em casos de atraso, a média foi de 12 sinais. Na comparação da relação de presença e ausência dos sinais entre os grupos com e sem atraso na aquisição da linguagem, oito sinais do bebê e um da mãe diferiram estatisticamente na amostra. O fator materno apresentou-se tão importante quanto o infantil na compreensão do funcionamento de linguagem dos bebês na análise realizada com o SEAL nos casos de atraso.

**Conclusão:**

Houve correlação significativa entre o desempenho no SEAL entre 3 e 24 meses e o desfecho de linguagem aos 24 meses avaliado pela Escala Bayley III nesta amostra.

## INTRODUÇÃO

A aquisição da linguagem infantil deve ser avaliada nos primeiros anos de vida do bebê, desde que haja um acompanhamento especializado na puericultura que contemple uma análise deste aspecto do desenvolvimento. Um dos instrumentos que permite uma avaliação nos dois primeiros anos de vida é a Escala Bayley III^(1)^, considerada padrão-ouro para avaliação do desenvolvimento infantil, amplamente utilizada pela comunidade científica^(2-10)^ por diferenciar a comunicação receptiva (49 itens) da expressiva (48 itens) no domínio gramatical infantil.

Na pesquisa de Madaschi et al.^(11)^ sobre a adaptação transcultural e propriedades psicométricas, a versão brasileira da escala Bayley III apresentou alta validade convergente e boa consistência interna e homogeneidade de itens para crianças de 12 a 42 meses, corroborando sua efetividade para fins de pesquisas.

Embora possua valor diagnóstico para o domínio gramatical, a referida escala demanda tempo de aplicação (de uma a duas sessões) e depende da colaboração da criança, bem como formação especializada do examinador e aquisição de materiais de alto custo, ao considerar a realidade habitual dos profissionais no Brasil. Além disso, não investiga a participação do adulto no processo de aquisição da linguagem.

Com o propósito de oferecer um instrumento que abordasse o diálogo adulto-criança e de fácil aplicação no processo de acompanhamento da aquisição da linguagem, foram validados preliminarmente os Sinais Enunciativos de Aquisição da Linguagem - SEAL^(12-14)^, que consideraram as contribuições de estudos clínicos na perspectiva enunciativa^(15-18)^ e estudo enunciativo de aquisição da linguagem^(19)^. Os sinais do SEAL consideram o nível semiótico (domínio gramatical da língua) e o processo de semantização da língua. Esse processo relaciona-se à apropriação que o sujeito faz de seu conhecimento linguístico (nível semiótico) na sustentação do diálogo, que permite que se identifique a emergência e sustentação de um lugar de enunciação para o bebê^(16-19)^.

Nessa perspectiva, são importantes as potencialidades da criança (biológicas, cognitivas e subjetivas) e a sustentação enunciativa que o adulto oferece^(17,18)^. Os sinais do SEAL captam se o processo de aquisição da linguagem está transcorrendo dentro do esperado ou se apresenta algum impedimento por meio de um paradigma indiciário, ou seja, se os sinais estão presentes possivelmente o processo está a contento, se ausentes, a criança e seus familiares devem ser acompanhados em consultas de menor intervalo de tempo para verificar o progresso em linguagem e estabelecer uma eventual demanda por intervenção oportuna. Os estudos do SEAL indicam um fator infantil e um fator materno no funcionamento de linguagem entre o bebê e sua mãe, que contribuem na compreensão de obstáculos à aquisição da linguagem^(12-14)^, demonstrando que é possível realizar intervenções oportunas para revertê-los a partir de uma escuta sensível da família. O SEAL não tem objetivo diagnóstico, mas de acompanhamento da aquisição da linguagem.

Assim, considerando os resultados obtidos de estudos anteriores sobre o domínio gramatical e processo de semantização da língua para analisar as condições da criança na ocupação do lugar de enunciação e o do adulto na sustentação desse lugar^(12-19)^, e as evidências científicas obtidas com a Escala Bayley III^(1-11)^, esta pesquisa analisa a correlação entre os resultados obtidos pelo SEAL e a Escala Bayley III, e compara bebês com e sem atraso na aquisição da linguagem aos 24 meses, em relação ao desempenho obtido por ele e sua mãe no SEAL dos 3 aos 24 meses.

## MÉTODO

Trata-se de um estudo quantitativo, longitudinal e prospectivo, aprovado pelo Comitê de Ética em Pesquisa de uma instituição de ensino em cidade de porte médio do Rio Grande do Sul, sob número de CAAE: 28586914.0.0000.5346. O estudo respeita as normas e diretrizes regulamentadoras para pesquisa com seres humanos que estão na Resolução 466/12 do Conselho Nacional de Saúde, prevendo a confidencialidade dos dados, garantindo sigilo e privacidade da identidade dos sujeitos, por meio da assinatura do Termo de Confidencialidade e do esclarecimento dos objetivos e procedimentos às famílias que assinaram o TCL (Termo de Consentimento Livre e Esclarecido) após terem aceitado participar da pesquisa. Os responsáveis pelos bebês responderam à entrevista sobre dados sociodemográficos, obstétricos e psicossociais adaptados na versão original de Schwengber e Piccinini^(20)^.

A amostra inicial na avaliação do SEAL foi de 101 bebês, mas destes apenas 45 crianças, 19 nascidas a termo e 26 pré-termo, acompanhadas pelo SEAL de 3 a 24 meses, foram avaliadas aos 24 meses pela Escala Bayley III. Nesta pesquisa será analisado o item linguagem. Os demais aspectos do desenvolvimento foram analisados a partir da Escala Bayley III por outros profissionais participantes da pesquisa maior. Para os bebês nascidos prematuros considerou-se a idade corrigida na avaliação. Os bebês e seus familiares foram convidados a participar da pesquisa no seguimento de prematuros de um hospital universitário e no teste do Pezinho em uma Unidade Básica de Saúde próxima ao local. Como critérios de inclusão buscou-se bebês sem limitações biológicas como lesões neurológicas ou síndromes, bem como déficits sensoriais (visual, auditivo, etc). Esses aspectos foram avaliados pelos pediatras, pela equipe de pesquisa que constou de fonoaudiólogos, psicólogos, fisioterapeutas e terapeutas ocupacionais. Em caso dúvida foram retirados da pesquisa e encaminhados para o neuropediatra ou geneticista.

Para análise de linguagem pelo SEAL foi realizada uma filmagem da interação da mãe ou quem desempenhava essa função para o bebê, que ocorreu de diferentes modos nas etapas do estudo. A filmagem foi realizada por dois ângulos: frontal e lateral, com tempo de 15 minutos, a depender da idade do bebê e demais aspectos a serem analisados. Os bebês foram posicionados em um bebê conforto ou sentados em tapete de EVA para interagir com suas mães, em ambiente iluminado e confortável em termos de temperatura. Deveriam estar em bom estado de vigília, bem alimentados e higienizados. A filmagem foi realizada com duas filmadoras JVC Everio GZ-MG 630 colocadas em duas posições: há dois metros da mãe e do bebê, pegando a mãe posicionada de costas para filmadora e o bebê de frente, e outra colocada há um metro, pegando a interação face a face da mãe com o bebê em ângulo lateral.

Houve uma padronização de posturas em que a criança foi observada na interação com sua mãe. Os bebês eram nascidos a termo e prematuros tardios. No caso deste grupo considerou-se a idade corrigida.

As faixas etárias em que os bebês foram filmados para análise do SEAL de acordo com os quatro instrumentos semestrais criados^(12-14)^ foram:

**Fase 1 - 3 meses e 1 dia a 4 meses e 29 dias** - O bebê sentado no bebê conforto (9 minutos). A mãe foi instruída a cantar (3 minutos) (ambientação), conversar com o bebê (3 minutos) e oferecer um objeto -ex- um cachorro de borracha sem barulho (3 minutos).
**Fase 2- 8 meses e 1 dia a 9 meses e 29 dias** - A mãe e bebê sentados no tapete EVA, foram filmados na interação, com a solicitação de a mãe cantar para o bebê por 3 minutos, conversar por mais 6 minutos e a brincar com um objeto (o cachorro de borracha) oferecido pelo examinador (6 minutos). Se o bebê não tivesse ainda domínio de tronco poderia utilizar bebê conforto.
**Fase 3 - 17 meses e um dia a 18 meses e 29 dias e Fase 4 - 23 meses e um dia a 24 meses e 29 dias** - Nestas fases o bebê foi observado em atividade livre com a mãe com uma caixa de brinquedos temáticos (animais, um bebê com mamadeira, panelinhas, etc.) e observou-se o brincar e a interação linguística entre mãe e bebê. A mãe foi orientada a permanecer sobre o tapete com o bebê durante a filmagem. Nos primeiros 10 minutos foi filmada a interação da mãe com o bebê e nos últimos 5 minutos o examinador participou da interação para observar alguns sinais que abrangiam o diálogo com distintos interlocutores.

Os vídeos foram assistidos por duas fonoaudiólogas habilitadas que atribuíram os sinais dos instrumentos do SEAL aos bebês, o que permitiu verificar uma concordância entre ambas 95 e 100%. Para a análise aqui apresentada foram considerados os valores atribuídos pela pesquisadora principal, primeira autora deste artigo.

Na Tabela 1 estão resumidos os sinais enunciativos analisados.

**Tabela 1:** Sinais Enunciativos de Aquisição da Linguagem (SEAL)

**Sinais de 2 a 6 meses e 29 dias**	**Locutor analisado**
1. A criança reage ao *manhês*, por meio de vocalizações, movimentos corporais ou olhar.	bebê
2. A criança preenche seu lugar na interlocução com sons verbais como vogais e/ou consoantes.	bebê
**3. A criança preenche seu lugar na interlocução com sons não verbais de modo sintonizado ao contexto enunciativo (sorriso, grito, choro, tosse, resmungo).**	bebê
**4. A criança preenche seu lugar na interlocução silenciosamente apenas com movimentos corporais e olhares sintonizados ao contexto enunciativo.**	bebê
5. A criança inicia a conversação ou protoconversação.	bebê
6. A criança e a mãe (ou sua substituta) trocam olhares durante a interação.	bebê-mãe
**7. A mãe (ou sua substituta) atribui sentido às manifestações verbais e não verbais do bebê, e sustenta essa protoconversação ou conversação, quando o bebê a inicia.**	mãe
8. A mãe (ou sua substituta) utiliza o *manhês* falando com a criança de modo sintonizado ao que está acontecendo no contexto e aguardando as respostas do bebê.	mãe
**Sinais de 7 a 12 meses e 29 dias**	
9. A criança preenche seu lugar na interlocução (enunciado) com sons verbais (sílabas com vogais e consoantes variadas - ao menos dois pontos e dois modos articulatórios de consoantes).	bebê
10. A criança esboça a produção de protopalavras por espelhamento à fala da mãe (ou substituto).	bebê
**11. A criança esboça a produção de protopalavras espontaneamente.**	bebê
**12. Quando a mãe (ou substituta) é convocada a enunciar pela criança, a mesma produz seu enunciado e aguarda a resposta da criança.**	mãe
**Sinais de 13 a 17 meses e 29 dias**	
**13. A criança nomeia de modo espontâneo e inteligível ao adulto interlocutor, objetos que estão ausentes no contexto.**	**bebê**
**14. A criança nomeia de modo espontâneo, mas não inteligível ao adulto interlocutor, objetos que estão ausentes no contexto, buscando na prosódia uma forma de ser compreendida.**	**bebê**
**15. A criança nomeia de modo espontâneo e inteligível ao adulto interlocutor, objetos, pessoas, ações, que estão presentes no contexto enunciativo.**	**bebê**
**16. A criança faz gestos para tentar fazer-se entender quando o adulto interlocutor não a compreende.**	**bebê**
**17. A criança repete o dizer do adulto interlocutor como forma de organizar ou reorganizar sua enunciação, por exemplo, aprimorando a forma sintática, ou fonológica, ou a escolha do item lexical ou mesmo acentuando algum item prosodicamente.**	**bebê**
**18. A criança conversa com diferentes interlocutores adultos (pai, mãe, examinador).**	**bebê**
**19. O adulto interlocutor atribui um sentido possível às produções verbais da criança, ou seja, de modo sintonizado.**	**mãe**
**Sinais 18 a 24 meses e 29 dias**	
**20. A criança solicita objetos e/ou pede esclarecimentos ao adulto interlocutor, marcando sua posição como locutor.**	**bebê**
**21. A criança utiliza formas fonêmicas distintas para veicular sentidos diferentes em sua enunciação (ao menos dois pontos articulatórios - labial e alveolar- e duas classes sonoras consonantais distintas - ao menos nasais e plosivas).**	**bebê**
**22. A criança utiliza distintas formas (palavras) para veicular sentidos diferentes em sua enunciação.**	**bebê**
**23. A criança combina palavras, na forma direta ou inversa, para veicular sentidos diferentes.**	**bebê**
**24. Quando a criança apresenta produções verbais distintas da fala adulta, o adulto interlocutor reage fazendo um pedido de reparo neutro (o que) ou repetindo corretamente a fala infantil ou oferecendo item lexical compatível com a intenção comunicativa do bebê.**	**mãe**

Fonte: Crestani et al.^(12,13)^, Fattore et al.^(14)^. Em negrito sinais significativos na análise fatorial

Todos os sujeitos também foram avaliados na fase 4 (faixa etária de 24 meses) por meio da Escala Bayley III^(1,11)^ por uma profissional habilitada para esta avaliação, sendo considerada nesta pesquisa a subescala de linguagem (comunicação receptiva e comunicação expressiva). Inicialmente foi encontrado o ponto de partida no teste de cada bebê, com base na sua idade. A avaliação teve início assim que o bebê pontuou consecutivamente as três primeiras questões (ponto base), e terminou com cinco erros seguidos.

Para a análise estatística utilizou-se um banco de dados em Excel em que foram organizados os dados de linguagem obtidos para presença e ausência de sinais do SEAL em cada faixa etária e total, bem como também os escores do Bayley III obtidos para 24 meses. Os resultados foram analisados estatisticamente no *software* STATISTICA 9.1. Para este estudo, considerou-se o nível de significância p ≤ 0,05. Foram utilizados os testes de correlação de Pearson e exato de Fisher.

## RESULTADOS

Foram analisados 45 bebês, foi verificada a correlação entre o número total de sinais do SEAL obtido por meio dos quatro instrumentos semestrais e os resultados de linguagem obtidos por meio da Escala Bayley III aos 24 meses. Os resultados pontuaram uma correlação **Pearson de 0,718 e um p_valor de 0,001**, indicando significância estatística (p<0,05). Isso permite concluir que quanto maior a pontuação do SEAL, maior a pontuação no Bayley III.

Na análise comparativa entre crianças com atraso de linguagem e sem atraso de linguagem por meio dos resultados obtidos na avaliação de linguagem na Escala Bayley III aos 24 meses, foi possível identificar o número médio de sinais enunciativos de aquisição da linguagem de cada grupo, conforme indicado na Tabela 2.

**Tabela 2:** Comparação pontuação total SEAL *versus* escala Bayley III

TESTES	SEM RISCO LINGUAGEM Bayley III 24 meses				COM RISCO LINGUAGEM Bayley III 24 meses				p_valor
	N	Média (±DP)	Mínimo	Máximo	N	Média (±DP)	Mínimo	Máximo	
SEAL TOTAL	25	18,85 (± 2,92)	15,00	22,00	20	12,39 (± 5,13)	2,00	22,00	0,001*

* Significativo pelo teste U de Mann-Whitney

**Legenda:** SEAL = Sinais Enunciativos de Aquisição da Linguagem; DP = Desvio Padrão; N = Número de Sujeitos

Observou-se que, em média, os bebês sem atraso na aquisição da linguagem apresentaram 18 sinais presentes, e que os bebês com atraso, 12 sinais, fato que se refletiu na diferença entre ambos grupos quando comparados a ausência e presença de cada sinal como se observa na Tabela 3.

**Tabela 3:** Análise comparativa por sinal dos bebês com e sem atraso em linguagem na escala Bayley III

SINAIS SEAL	Bebês com atraso n=20				Bebês sem atraso n=25				Comparação*
	Díades com sinal ausente		Díades com sinal presente		Díades com sinal ausente		Díades com sinal presente		
	n	%	n	%	N	%	n	%	p-valor
1 (B)	2	10	18	90	2	8	23	92	0,606
2 (B)	6	30	14	70	3	12	22	88	0,131
**3 (B)**	2	10	18	90	2	8	23	92	0,606
**4 (B)**	2	10	18	90	2	8	23	92	0,606
5 (B)	10	50	10	50	11	44	14	56	0,460
6 (B)	3	15	17	85	2	8	23	92	0,392
**7 (A)**	2	10	18	90	1	4	24	96	0,415
8 (A)	5	25	15	75	3	12	22	88	0,229
9 (B)	11	55	9	45	6	24	19	76	0,034*
10 (B)	13	65	7	35	7	28	18	72	0,014*
**11 (B)**	11	55	9	45	9	36	16	64	0,165
**12 (A)**	2	10	17	85	3	12	22	88	0,632
13 (B)	20	100	0	0	22	88	3	12	0,625
**14 (B)**	20	100	0	0	25	100	0	0	1,000
**15 (B)**	15	75	5	25	4	6	21	94	0,0001*
16 (B)	9	45	11	55	0	0	25	100	0,0002*
**17 (B)**	16	80	4	20	4	6	21	94	0,0000*
**18 (B)**	14	70	6	30	3	12	22	88	0,0001*
**19 (A)**	11	55	9	45	7	28	18	72	0,063
**20 (B)**	8	40	12	60	4	6	21	94	0,071
**21 (B)**	12	60	8	40	1	4	24	96	0,0000*
**22 (B)**	17	85	3	15	4	6	21	84	0,0000*
**23 (B)**	15	75	5	25	4	6	21	84	0,0001*
**24 (A)**	10	50	10	50	1	4	24	96	0,0005*

* Significativo teste exato de Fisher

**Legenda:** Negrito = sinais importantes na análise fatorial (Crestani et al.^(12,13)^, Fattore et al.^(14)^); B = Sinal do Bebê; A = Sinal do Adulto; n = Número de Bebês; SEAL = Sinais Enunciativos de Aquisição da Linguagem

Os sinais 9, 10, 16, 17, 18, 21, 22 e 23, referentes a aspectos enunciativos do bebê, e o sinal 24, relativo à posição materna na última faixa etária, diferenciaram o grupo com atraso na aquisição da linguagem do grupo sem atraso na comparação estatística da amostra. Os bebês com desenvolvimento típico tiveram maior presença desses sinais do que os bebês com atraso.

A análise descritiva dos dados das 20 crianças que apresentaram alteração de linguagem pela Escala Bayley III, aos 24 meses, está descrita na Tabela 4, com a indicação dos sinais presentes e ausentes.

**Tabela 4:** Análise descritiva dos Sinais Enunciativos de Aquisição da Linguagem nos casos de atraso em linguagem no Bayley III aos 24meses

Sujeito	Sinais Presentes SEAL	Sinais Ausentes SEAL	Total SEAL Presentes	Bayley III 24m
S1	1,2,3,4,5,6,7,8,12,16,18,19,20, 21,24	9,10,11, 13, 14, 15,17, 22,23	15	79 (L)
S2	1,3,4,5,6,7,8,16,18,	2,9,10,11,12, 13,14,15,17 19, 20,21,22,23,24	9	71 (L)
S3	1,2,3,4,5,6,7,8,9,10,11,12,	13,14,15,16,17,18,19,2021,22,23, 24	12	59 (EB)
S4	1,3,4,6,7,8,12, 16,19	2,5,9,10,11,13,14,15,17,18,20,21,22,23,24	9	77 (L)
S5	1,2,3,4,5,6,7,8,9,10,11,12,15,16,18,19,20,21,24	13,14,17,22,23	19	83 (MB)
S6	1,2,3,4,5,6,7,8,12, 20,21,24	9,10,11,13,14,15,16,17, 18,19, 22,23	12	79 (L)
S7	6,12	1,2,3,4,5,7,8,9,10,11,13, 14,15,16,17,18,19,20,21,22,23,24	2	65 (EB)
S8	1,3,4,6,7,8,12,20,21,22,23,24	2,5,9,10,11,13,14,15,16, 17,18,19	12	47 (EB)
S9	1,2,3,4,5,6,7,8,9,10,11,12,16,19,20,21,22	13,14,15,17,18,23,24	17	85 (MB)
S10	1,2,3,4,7,11,12,	5,6,8,9,10,13,14,15,16,17,18,19,20,21,22,23,24	7	59 (EB)
S11	1,2,3,4,5,6,10,11,12,20,21,22,23	7,8,9,13,14,15,16,17,18,19,24	13	74 (L)
S12	1,2,3,4,5,6,7,8,9,10,11,12,15,16,17,18,19,20,21,22,23,24	13,14	22	77 (L)
S13	1,2,3,4,7,9,11	5,6,8,10,12,13,14,15,16,17,18,19,20,21,22,23,24	7	47 (EB)
S14	2,7,8,15,16,17,18,19,20,21,22,24	1,3,4,5,6,9,1011,12,13,14,23	12	79 (L)
S15	1,3,4,6,7,12,16,20,21,24	2,5,8,9,1011,13,14,15,17,18,19,22,23	10	79 (L)
S16	1,2,3,4,5,6,7,8,9,10,11,12,16,	13,14,15,17,18,19,20,21,22,23,24	13	77 (L)
S17	1,2,3,4,6,7,8,9,12,15,16,17,18,19,20,21,22,23,24	5,10,11,13,14	19	79 (L)
S18	1,2,3,4,5,6,7,8,9,10,11,12,15,16,17,18,19,20,21,22,23,24	13,14	22	83 (MB)
S19	1,3,4,6,7,8,12,13,20,21	2,5,9,10,11,14,15,16,17,18,19, 22,23,24	10	83 (MB)
S20	1,2,3,4,6,7,8,9,12,20,21,22,23,24	5,10,11,13,14,15,16,17,18,19	14	89 (MB)

**Legenda:** L = Limítrofe; EB = Extremamente Baixa; MB = Média Baixa; SEAL = Sinais Enunciativos de Aquisição da Linguagem

Observou-se que as crianças com alterações pelo SEAL tiveram pontuação limítrofe, baixa ou muito baixa pela Escala Bayley III. As crianças que desenvolveram o atraso na avaliação pela Escala Bayley III aos 24 meses, apresentaram alterações em sinais da criança (fator infantil) e nos sinais relativos à atividade materna de sustentação do diálogo (fator materno). Ainda, deste grupo de 20 crianças com alteração pela Escala Bayley III, quatro não obtiveram risco no SEAL, de acordo com o número médio de sinais (Tabela 2), bem como não apresentaram alterações do fator materno (sinais em negrito). As quatro crianças sem risco pelo SEAL, mas com atraso pela Escala Bayley III, apresentaram avaliações entre limítrofe e média baixa. Nenhuma delas recebeu a atribuição de extremamente baixa.

Outro aspecto relevante na Tabela 4 é a variação na pontuação obtida na Escala Bayley III (limítrofe, muito baixa, extremamente baixa) que ressalta a maior parte das crianças na categoria extremamente baixa, mais indicativa de grave atraso, com os menores valores no SEAL (2 a 7) na exceção do sujeito 3 (S3) com 12 sinais presentes, ainda um valor baixo, o que evidencia a concordância ao menos nos aspectos referentes ao fator infantil entre ambos testes. As crianças com as outras categorias (limítrofe e muito baixa) pontuaram valores entre 9 e 17 sinais no SEAL.

## DISCUSSÃO

A correlação positiva entre casos com atraso de linguagem avaliados pela Escala Bayley III e casos de risco pelo SEAL permite afirmar que este teste foi efetivo como teste de triagem na amostra estudada. Na Tabela 4, observou-se que 16 dos 20 casos avaliados com atraso pela Escala Bayley III, estiveram com alteração importante no SEAL.

Nesta pesquisa, o SEAL foi atribuído por filmagens, em acompanhamentos de puericultura, embora possa ser analisado pelo profissional habilitado em sua formação, durante uma observação da interação mãe-bebê em ambulatório, o que seria menos dispendioso em termos de tempo e custo para inserção no SUS. Nesta condição, os casos suspeitos pelo SEAL seriam enviados para um teste diagnóstico com a Escala Bayley III ou outras escalas diagnósticas em linguagem e desenvolvimento^(21,22)^. Cabe destacar que o número de díades mãe-bebê participantes na investigação do SEAL em todas as faixas etárias foi muito superior (101) ao grupo que compareceu aos dois encontros de avaliação da Escala Bayley III (45). Essa perda amostral sugere que a adesão ao realizar o teste mais demorado (a Escala Bayley III) por parte dos usuários demanda uma mudança de cultura e melhoria no acesso ao serviço de saúde, algo que não se vislumbra mudar em uma perspectiva de curto prazo.

Já a comparação realizada entre o grupo com atraso e sem atraso permitiu estabelecer uma média de 18 sinais enunciativos, entre os 24 avaliados, como ausência de risco. Esse dado sugere a necessidade de dar continuidade na investigação do SEAL em termos de estabelecimento de critérios por faixa etária e no total do teste, o que não foi possível com a pequena amostra obtida nesta pesquisa.

Na Tabela 3, observa-se que há sinais como o 13 e 14 que estiveram ausentes em ambos grupos, embora o sinal 14 tenha sido indicado como relevante na análise fatorial^(14)^. Entre os sinais que diferiram estatisticamente na comparação entre bebês com atraso e sem atraso, os Sinais 9, 10, 21, 22 e 23 evidenciam habilidades de o bebê sem atraso na linguagem de ocupar seu lugar de enunciação com vocalização e fala crescentemente mais complexas (diversidade fonológica e lexical, e uso inicial de sintaxe). Já bebês sem esses sinais pode evidenciar o possível atraso na aquisição da linguagem.

O sinal 16 se relaciona, por outro lado, ao uso da gestualidade como forma de comunicação, o que é previsto por estudos de aquisição da linguagem que afirmam haver uma continuidade e sincronia entre habilidades verbais e gestualidade do bebê^(23)^.

O sinal 17 relaciona-se à capacidade de o bebê se ancorar na fala da mãe para aprimorar o seu dizer, uma estratégia identificada por Silva^(19)^ no processo de aquisição da linguagem. Ela também está relacionada a uma disposição do adulto em socorrer a criança em seu dizer, avaliada no sinal 24. Portanto, é importante observar a força de ambos sinais simultaneamente na amostra estudada.

Além deles, o sinal 18 que se relaciona à amplitude de interlocutores, é fundamental na avaliação não só da disjunção em termos de relação enunciativa de aquisição, mas também do processo de separação mãe-bebê, como visto no estudo de Flores e Souza^(24)^, em que bebês em sofrimento psíquico e dificuldades no processo de separação e operação da função paterna, evidenciaram dificuldade de falar com distintos interlocutores.

O fato de nenhum sinal ter sido distintivo na comparação entre ambos grupos na primeira faixa etária (zero a seis meses) indica a necessidade de continuar investigando e aprimorando o instrumento, visto que a análise fatorial demonstrou três sinais nessa fase relativos a um fator infantil e um materno em uma amostra maior de sujeitos nesta fase^(13)^. Esses resultados indicam a necessidade de continuar os estudos de validação de critério.

Nos dados observados na Tabela 4, cabe destacar que os sujeitos 5, 12, 17 e 19, apresentaram 18 ou mais sinais enunciativos presentes. Cumpre ressaltar que esses quatro sujeitos não apresentaram alterações no fator materno, assim como o sujeito 5 com atraso pelo SEAL e pela Escala Bayley III, enquanto todos os demais sim. Eles também não obtiveram a classificação “extremamente baixa” na Escala Bayley III^(1)^. Esse dado permite observar que, na maior parte dos casos, houve a contribuição do fator materno para a emergência e compreensão do funcionamento de linguagem nos casos de atraso na aquisição da linguagem, ou seja, a forma como o adulto realiza a sustentação enunciativa é um aspecto que deve ser considerado na avaliação e intervenção junto aos atrasos de aquisição da linguagem^(17,18)^.

Diversos estudos do campo enunciativo^(12-19)^ têm evidenciado que tanto a aquisição da linguagem, quanto a clínica com crianças pequenas, devem investir na análise do diálogo mãe-bebê para propor uma hipótese de funcionamento de linguagem^(16)^ que permita propor linhas de intervenção. Essa hipótese de funcionamento de linguagem prevê a relação EU (criança) - TU (adulto) no entendimento do sofrimento advindo do atraso ou distúrbio de linguagem. A partir dessa perspectiva teórica e dos resultados observados nesta pesquisa, observou-se que o SEAL é promissor por avaliar tal fator cuja importância foi identificada na presença do mesmo em 16 dos 20 casos avaliados como atraso de linguagem pela Escala Bayley III. Destaca-se a importância de que crianças com valores extremamente baixos pela Escala Bayley III também foram as com menores valores no SEAL.

Considerando a limitação numérica da amostra deste estudo, os resultados encontrados sugerem a necessidade de continuar investigando a linguagem de bebês e crianças pequenas com o SEAL, pois realça um modo efetivo de acompanhar a aquisição da linguagem na puericultura e propor intervenções a tempo de impedir a cristalização de sintomas de linguagem^(17,18)^.

Para tanto, será necessário estabelecer critérios para o teste em amostras maiores, visto que este foi um estudo clínico, de menor proporção, com os bebês que compareceram à avaliação realizada com a Escala Bayley III no desfecho da pesquisa aos dois anos, número muito inferior ao desejado. As avaliações com o SEAL não possuem fins diagnósticos, uma vez que o bebê está em processo de constituição linguística, mas buscam oferecer intervenções oportunas que possam favorecer um encontro entre os familiares e o bebê ou criança pequena, pois facilitar o fator materno, que se apresentou importante nos estudos fatoriais, é uma forma de fortalecer o encontro e sincronia linguística entre a mãe (ou sua substituta) e o bebê^(13-15)^. Isso evidencia que a Fonoaudiologia poderia se beneficiar de estudos que tomem o diálogo como foco da análise nas pesquisas de aquisição da linguagem, pois as habilidades infantis para ocupar seu lugar de enunciação são tão relevantes no processo de aquisição, quanto a sustentação que o adulto faz de um lugar de enunciação.

## CONCLUSÃO

É possível concluir que houve correlação significativa entre o desempenho no SEAL entre 3 e 24 meses e o desfecho de linguagem aos 24 meses avaliado pela Escala Bayley III. A comparação entre bebês com e sem atraso na aquisição da linguagem permitiu estabelecer médias de sinais no SEAL e em alguns sinais do bebê e um da mãe apresentaram diferenças estatísticas na comparação dos dois grupos da amostra estudada quanto a sua presença e ausência, sobretudo a partir da segunda faixa etária estudada.

Tais dados permitem afirmar que o SEAL tem potencial como teste de triagem e deve ser investigado em amostras maiores, pois é rápido e exige apenas a observação da interação mãe-bebê no primeiro e segundo anos de vida, em meio a uma brincadeira naturalista com materiais acessíveis aos examinadores e às famílias.
